# Effects of aerobic exercise on tear secretion and tear film stability in dry eye patients

**DOI:** 10.1186/s12886-021-02230-9

**Published:** 2022-01-04

**Authors:** Chao Sun, Xiaofan Chen, Yanming Huang, Huan Zou, Wei Fan, Mei Yang, Rongdi Yuan

**Affiliations:** Department of Ophthalmology, the Second Affiliated Hospital of Army Medical University, 183#, Xinqiaozheng St., Shapingba District, Chongqing, 400037 People’s Republic of China

**Keywords:** Dry eye, Aerobic exercise, Schirmer I test, Tear film stability, Non-invasive tear breakup time, Blinking

## Abstract

**Background:**

To study the effects of aerobic exercise (AE) on tear secretion and tear film stability in dry eye patients.

**Methods:**

This study consisted of two parts, each part included 3 groups, namely dry eye without AE group, dry eye with AE group and pre-clinical dry eye with AE group. In part 1, we studied the variations of Schirmer I test and six tear compositions before and after AE (34 eyes in each group). In part 2, we studied the variations of tear meniscus height, first and average non-invasive tear breakup time (F-NITBUT and A-NITBUT), lipid layer thickness, number of incomplete and complete blinks, partial blink rate (PBR) and visual acuity before and after AE (30 eyes in each group).

**Results:**

In dry eye with AE group, Schirmer I test at 0 min after AE increased significantly compared to baseline (*P* < 0.001), the oxidative stress marker 8-hydroxy-2′-deoxyguanosine after AE decreased significantly compared to baseline (*P* = 0.035, *P* = 0.045), F-NITBUT and A-NITBUT after AE prolonged significantly compared to baseline (*P* < 0.001, *P* = 0.007, *P* = 0.036; *P* < 0.001, *P* = 0.001, *P* = 0.044), number of incomplete blinks and PBR at 10 min after AE decreased significantly compared to baseline (*P* < 0.001; *P* < 0.001) while number of complete blinks increased significantly (*P* < 0.001). Besides, significant differences were also found between dry eye with AE group and dry eye without AE group at all above corresponding time point (*P* < 0.05).

**Conclusion:**

AE promotes tear secretion and improves tear film stability in dry eye patients. AE may be a potential treatment for dry eye.

**Trial registration:**

Chinese Clinical Trial Registry, ChiCTR2000038673. Registered 27 September 2020,

**Supplementary Information:**

The online version contains supplementary material available at 10.1186/s12886-021-02230-9.

## Background

Dry eye is a multifactorial ocular surface disease characterized by a loss of homeostasis of the tear film. The main pathophysiological mechanisms include tear film instability, hyperosmolarity and ocular surface inflammation and damage [1]. Dry eye has a significant impact on vision function and quality of life. Dry eye prevalence ranges from 5 to 50% globally, it increases with age [2] and the widespread use of visual display terminals [3]. Currently, drug therapy is the main treatment for dry eye. Drug therapy restores the microenvironment of the ocular surface by promoting tear secretion and anti-inflammatory treatment [4]. However, due to the complex etiology and poor consistency between signs and symptoms [5], the drugs have limited effects [6]. For moderate and severe dry eyes, drug treatment can only relieve some of the symptoms while it is not suitable for long-term use. Therefore, more new non-drug treatment strategies need to be explored.

Aerobic exercise (AE) is an effective treatment for many systemic diseases especially chronic diseases [7]. Currently, animal studies have shown that 8 weeks of AE increases tear secretion in diabetic mice [8]. A large population study found that lack of exercise was closely related to increased susceptibility to dry eye [3]. Ten weeks of exercise improves the subjective symptoms of dry eye patients [9]. The aim of this study was to determine the specific effects and possible mechanisms of AE on tear secretion and tear film stability.

## Methods

### Subjects

This research was implemented in accordance with the requirements of the Declaration of Helsinki and the protocol was approved by the Ethics Committee of the Second Affiliated Hospital of Army Medical University. The clinical trial registration number is ChiCTR2000038673.

All subjects were 18–30 years old and agreed to participate in this study and signed an informed consent. The diagnostic criteria for dry eye and pre-clinical dry eye were as follows: Symptoms and positive result of non-invasive tear breakup time constitute the diagnosis of dry eye. The screening Ocular Surface Disease Index (OSDI) confirmed that a patient might have dry eye and triggered diagnostic testing of non-invasive tear breakup time. Positive result were OSDI score ≥ 13 and average non-invasive tear breakup time (A-NITBUT) < 10s according to the Tear Film and Ocular Surface Society Dry Eye Workshop II (DEWS II) [10]. Symptomatic patients without demonstrable clinical signs were differentiated into pre-clinical dry eye [1]. Subjects were excluded if they had neuropathic pain, allergic conjunctivitis, Sjögren’s syndrome, lacrimal obstruction or other diseases. Subjects were also excluded if they had dry eye treatment or used contact lenses within 1 month, if they had a history of eye trauma or surgery within 1 year or subjects with serious systemic diseases who were not suitable for strenuous exercise.

### Study design

The test was divided into two parts in order to avoid the possible influences of Schirmer I test on the subsequent measurements of signs associated with dry eye. Each part included 3 groups, namely dry eye without AE group, dry eye with AE group and pre-clinical dry eye with AE group. In part 1, we studied the variations of Schirmer I test and six tear compositions before and at 0, 30 min after AE. The tear compositions tested included dry eye diagnostic factor lactoferrin [11] and matrix metalloproteinase-9 (MMP-9) [12], dry eye inflammation marker IL-6 [13], oxidative stress marker 8-hydroxy-2′-deoxyguanosine (8-OHdG) [14], (O-acyl)-ω-hydroxy fatty acids (OAHFA) which is closely related to tear film stability [15], and Mucin 5 subtype AC (MUC5AC) which is considered to be the most abundant secretory mucin in human tears [16]. In part 2, we studied the variations of signs associated with dry eye before and at 10, 20, 40 min after AE including tear meniscus height (TMH), first non-invasive tear breakup time (F-NITBUT), A-NITBUT, tear film lipid layer thickness (LLT), incomplete and complete blinks and partial blink rate (PBR), and the variations of visual acuity before and at 0, 30, 60 min after AE. The number of subjects in each group are reported in Table 1. During the test period, all the subjects were exposed to the same environment, and they were required to fast because eating have an effect on tear secretion [17].

**Table 1:** Comparison of basic information

		Dry eye without AE	Dry eye with AE	Pre-clinical dry eye with AE
Part 1	NO. subjects (eyes)	17 (34)	17 (34)	17 (34)
	Male (eyes): Female (eyes)	12 (24): 5 (10)	12 (24): 5 (10)	12 (24): 5 (10)
	Age (years)	22.0 (21.0, 23.5)	22.0 (21.0, 24.0)	21.0 (21.0, 23.3)
	OSDI score	25.0 (19.4, 32.2)	22.7 (16.0, 32.2)	16.7 (7.3, 21.3)^a^
Part 2	NO. subjects (eyes)	15 (30)	15 (30)	15 (30)
	Male (eyes): Female (eyes)	10 (20): 5 (10)	12 (24): 3 (6)	12 (24): 3 (6)
	Age (years)	21.0 (21.0, 23.0)	22.0 (21.0, 23.3)	21.0 (21.0, 23.0)
	OSDI score	22.9 (20.5, 32.5)	25.0 (20.0, 45.0)	13.6 (5.0, 25.0)^a^

^a^ There were significant differences compared with the other groups (*P* < 0.05)

*OSDI* the ocular surface disease index

### AE protocol

The venue was outdoors, the ambient temperature was 25–27 °C while the humidity was 50–60% during the test. The measuring time was between 18:00 and 19:00. The AE protocol was defined as jogging for 30 min. According to the 6–20 Rating of Perceived Exertion Scale, the target heart rate was set to 64–76% of the maximum heart rate in order to achieve moderate exercise intensity [18]. The maximum heart rate was defined as 220 minus age [18].

### Test items

#### OSDI

The Ocular Surface Disease Index (OSDI) questionnaire was used to quantify the subjective symptoms of dry eye [10]. The full questionnaire is available as a supplementary file (online supplementary file 1). The symptoms and environmental triggers for dry eye in the past week were assessed. OSDI score ≥ 13 was considered positive. The score range was 0–100. The higher the score, the more severe the symptoms.

#### Schirmer I test

The researcher wore gloves and placed the Schirmer test strip (35 mm; DSA Exports, India, without fluorescent agent) at the outer 1/3 of the lateral eyelid margin without using anesthetics. Both eyes were tested simultaneously and the eyes remained closed during the procedure. The strip was removed after 5 min and the length of wetted part up to the indentation line was recorded. Each test was carried out in a quiet and dark environment.

### Analysis of tear compositions

Schirmer test strips were used to collect the tears. The test strips were stored in a refrigerator at − 80 °C, and placed in a refrigerator at 2–4 °C overnight before testing. Amount of PBS buffer to be added to each strip was calculated by multiplying the wetted length of the Schirmer test strip (Schirmer test reading + about 5 mm of the head of strip without scale) by 100 μl. After homogenizing, the samples were centrifuged at 2–4 °C for 15 min (2000 rpm). In total, 10 μl of the supernatant was collected for analysis after precipitation. Six compositions of the tear were determined using ELISA kit (Jiangsu Meimian, China). The absorbance was measured at 450 nm with a multifunctional microplate reader (Labsystems Multiskan MS, Finland).

### Measurements of signs associated with dry eye

Keratograph 5 M (OCULUS, Wetzlar, Germany) was used to measure TMH, F-NITBUT and A-NITBUT. Images with unclear boundaries of the tear meniscus were deleted to avoid affecting image analysis. The F-NITBUT defined as the time in each measurement between the last complete blink and the first perturbation or irregularity of the rings of the Placido disc reflected on the corneal surface. The A-NITBUT defined as the average of all tear film breakups occurring in the measured period in each measurement. The measurement was performed twice by the same ophthalmologist and the mean was taken. LipiView LVI-1001 (TearScience, Inc., Morrisville, North Carolina) was used to measure LLT and shot 20s video which automatically recorded incomplete and total blinking [19]. Number of complete blinks (number of total blinks minus incomplete blinks) and PBR (number of incomplete blinks / total blinks) were obtained using simple calculation.

### Visual acuity

ETDRS chart was used to measure the best corrected visual acuity.

### Statistical analysis

Statistical analyses were performed with SPSS version 20.0 software package (IBM Corp., Armonk, NY, USA). Data was expressed as median (25% interquartile, 75% interquartile) or mean ± standard deviation. The Mann-Whitney *U* test was applied to compare the age and OSDI score between two groups. The Schirmer I test, tear compositions, signs associated with dry eye and visual acuity at different time points were compared using two-way repeated measures analysis of variance. Bonferroni correction was used for multiple comparisons. The level of significance was set at *P* < 0.05.

### Sample size

The sample size was calculated using PASS software (version 15.0, NCSS, LLC). According to the results of the A-NITBUT in preliminary experiment, the sample size was estimated by adopting the significance level (α) as 0.05, the desired power (1-β) as 0.85, the autocorrelation coefficient as 0.7 and the standard deviation of the population of 5.1. The estimated sample size was at least 29 eyes in each group.

## Results

Age and Sex distribution and OSDI score of each part were mentioned in Table 1. The heart rate of all subjects reached the target and none of those subjects experienced any discomfort or pain during the examination.

### Changes of tear volume and tear compositions before and after AE

**Fig. 1:**
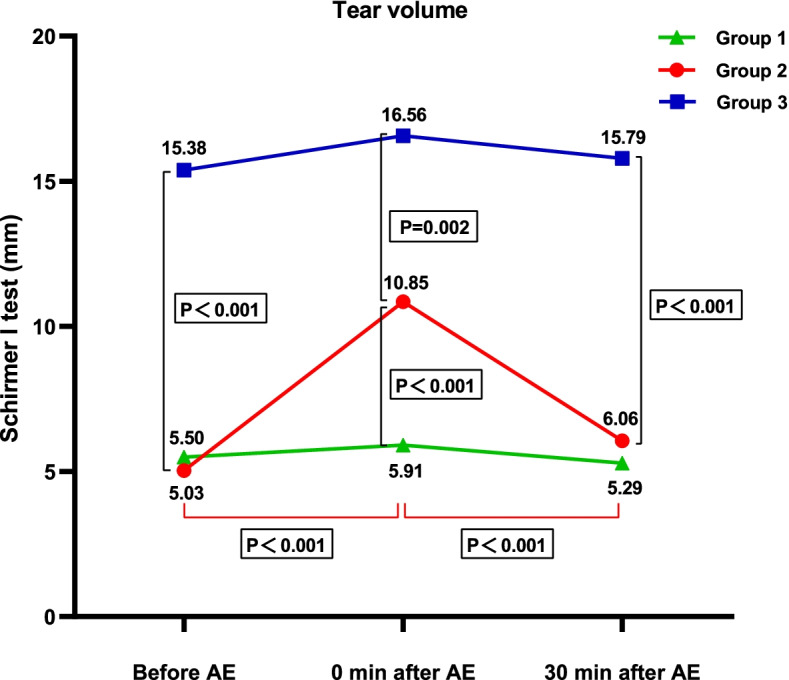
Comparison of tear volume before (baseline) and after AE. Group 1: Dry eye without AE, Group 2: Dry eye with AE, Group 3: Pre-clinical dry eye with AE

Tear volume was evaluated using Schirmer I test. In dry eye with AE group, tear volume at 0 min after AE was significantly increased compared to baseline (*P* < 0.001), and was significantly higher than dry eye without AE group (*P* < 0.001). Tear volume decreased significantly at 30 min after AE compared to 0 min after AE (*P* < 0.001) (Fig. 1).

In dry eye with AE group, 8-OHdG were significantly decreased in 2 measurements after AE compared to baseline (*P* = 0.035, *P* = 0.045), and were significantly lower than dry eye without AE group (*P* = 0.005, *P* = 0.018). There were no significant changes in the other tear compositions before and after AE (*P* > 0.05) (Table 2).

**Table 2:** Comparison of tear compositions before (baseline) and after AE

	Group	Time			*P value*			
		Baseline	0 min	30 min		Group ×Time	Group main effect	Time main effect
Lactoferrin (μg/ml)	1	110.08 ± 16.02	113.51 ± 12.28	114.69 ± 13.04	1 and 2	0.840	0.656	0.068
	2	111.63 ± 15.14	115.72 ± 15.46	114.88 ± 13.06				
					2 and 3	0.535	0.038	0.706
	3	120.04 ± 16.92	120.24 ± 16.50	118.46 ± 16.26				
MMP-9 (μg/L)	1	1071.16 ± 166.35	1058.28 ± 170.00	1059.03 ± 172.75	1 and 2	0.156	0.165	0.072
	2	1065.60 ± 157.43	984.20 ± 151.56	999.36 ± 171.43				
					2 and 3	0.652	0.041	0.097
	3	976.62 ± 178.87	940.23 ± 190.27	959.15 ± 171.21				
IL-6 (ng/L)	1	19.63 ± 2.05	19.49 ± 1.87	19.51 ± 2.02	1 and 2	0.221	0.185	0.107
	2	19.52 ± 2.01	18.50 ± 1.96	18.96 ± 2.16				
					2 and 3	0.286	0.069	0.377
	3	18.42 ± 1.82	18.44 ± 2.07	18.60 ± 2.30				
8-OHdG (ng/L)	1	105.17 ± 10.77	105.67 ± 10.95	105.62 ± 11.24	1 and 2	0.016	/	/
	2	105.43 ± 10.74	98.00 ± 11.74^ab^	98.23 ± 13.03^ab^				
					2 and 3	0.142	0.229	0.031
	3	99.32 ± 12.84	95.65 ± 11.08	100.16 ± 12.20				
OAHFA (pg/ml)	1	367.21 ± 40.58	366.66 ± 35.39	371.12 ± 32.89	1 and 2	0.704	0.842	0.904
	2	365.13 ± 43.50	369.71 ± 38.53	365.75 ± 36.82				
					2 and 3	0.918	0.879	0.792
	3	362.77 ± 35.43	366.95 ± 40.08	368.28 ± 42.66				
MUC5AC (μg/L)	1	92.65 ± 8.10	93.50 ± 8.21	93.83 ± 8.22	1 and 2	0.945	0.741	0.736
	2	93.54 ± 8.17	93.65 ± 9.24	94.34 ± 8.24				
					2 and 3	0.412	0.019	0.492
	3	98.89 ± 9.81	95.35 ± 7.78	96.26 ± 8.13				

Group 1: Dry eye without AE, Group 2: Dry eye with AE, Group 3: Pre-clinical dry eye with AE

^a^ There was a significant difference compared with baseline (*P* < 0.05)

^b^ There was a significant difference compared with Group 1 (*P* < 0.05)

*MMP-9* matrix metalloproteinase-9, *8-OHdG* 8-hydroxy-2′-deoxyguanosine, *OAHFA* (O-acyl)-ω-hydroxy fatty acids, *MUC5AC* Mucin 5 subtype AC

### Changes in signs associated with dry eye before and after AE

In dry eye with AE group, F-NITBUT and A-NITBUT were significantly prolonged in 3 measurements after AE compared to baseline (*P* < 0.001, *P* = 0.007, *P* = 0.036; *P* < 0.001, *P* = 0.001, *P* = 0.044), and were significantly longer than dry eye without AE group (*P* < 0.001, *P* = 0.004, *P* = 0.004; *P* < 0.001, *P* = 0.001, *P* = 0.005). The number of incomplete blinks and PBR were significantly decreased at 10 min after AE compared to baseline, 20 and 40 min after AE (*P* < 0.001, *P* = 0.013, *P* = 0.022; *P* < 0.001, *P* = 0.002, *P* < 0.001), and were significantly lower than dry eye without AE group (*P* < 0.001; *P* < 0.001), while the number of complete blinks was significantly increased (*P* < 0.001, *P* = 0.002, *P* < 0.001), and was significantly higher than dry eye without AE group (*P =* 0.001). TMH, LLT and number of total blinks had no significant difference before and after AE (*P* > 0.05) (Table 3).

**Table 3:** Comparison of signs associated with dry eye before (baseline) and after AE

	Group	Time				*P value*			
		Baseline	10 min	20 min	40 min		Group × Time	Group main effect	Time main effect
TMH (mm)	1	0.31 ± 0.07	0.30 ± 0.09	0.30 ± 0.07	0.30 ± 0.06	1 and 2	0.984	0.784	0.630
	2	0.32 ± 0.13	0.31 ± 0.11	0.31 ± 0.13	0.30 ± 0.09				
						2 and 3	0.404	0.866	0.537
	3	0.31 ± 0.09	0.30 ± 0.07	0.30 ± 0.09	0.32 ± 0.11				
F-NIT BUTF-NITBUT (s)	1	5.52 ± 1.97	5.36 ± 1.68	5.44 ± 1.54	5.44 ± 1.35	1 and 2	<0.001	/	/
	2	5.44 ± 1.88^c^	10.80 ± 5.35^ab^	7.94 ± 3.88^ab^	7.87 ± 4.24^ab^				
						2 and 3	0.036	/	/
	3	10.16 ± 4.07	10.77 ± 5.66	9.50 ± 5.48	9.64 ± 5.05				
A-NIT BUTA-NITBUT (s)	1	7.49 ± 2.36	7.91 ± 2.04	7.91 ± 2.18	7.83 ± 1.39	1 and 2	0.001	/	/
	2	7.73 ± 1.63^c^	12.94 ± 4.65^ab^	11.04 ± 4.41^abc^	10.34 ± 4.83^abc^				
						2 and 3	0.003	/	/
	3	14.37 ± 3.42	14.67 ± 5.56	13.70 ± 5.41	13.73 ± 5.48				
LLT (nm)	1	48.23 ± 16.66	50.33 ± 18.70	49.10 ± 15.10	46.17 ± 16.18	1 and 2	0.363	0.946	0.117
	2	52.37 ± 22.05	48.27 ± 18.85	50.17 ± 17.55	45.23 ± 16.40				
						2 and 3	0.424	0.003	0.086
	3	63.43 ± 20.71	56.93 ± 21.00	57.43 ± 18.17	59.27 ± 15.27				
Number of incomplete blinks	1	4.33 ± 3.06	4.43 ± 3.57	4.17 ± 3.03	4.30 ± 3.41	1 and 2	0.033	/	/
	2	4.30 ± 3.41^c^	1.60 ± 1.45^bcd^	3.17 ± 2.25	3.47 ± 3.20^c^				
						2 and 3	<0.001	/	/
	3	2.77 ± 1.83	2.77 ± 2.03	2.80 ± 1.97	2.37 ± 1.99				
Number of complete blinks	1	4.00 ± 2.95	4.57 ± 2.36	4.60 ± 1.96	4.50 ± 2.58	1 and 2	<0.001	/	/
	2	3.80 ± 2.83	7.90 ± 4.87^bcd^	4.93 ± 2.59	4.70 ± 2.77				
						2 and 3	<0.001	/	/
	3	4.33 ± 2.40	4.13 ± 2.91	3.87 ± 2.73	3.60 ± 2.14				
Number of total blinks	1	8.33 ± 4.90	9.00 ± 4.22	8.77 ± 4.08	8.80 ± 4.56	1 and 2	0.690	0.754	0.353
	2	8.10 ± 5.16	9.50 ± 5.22	8.10 ± 3.49	8.17 ± 4.50				
						2 and 3	0.325	0.035	0.106
	3	7.10 ± 3.64	6.90 ± 3.88	6.67 ± 4.37	5.97 ± 3.32				
PBR	1	0.52 ± 0.23	0.47 ± 0.22	0.45 ± 0.17	0.49 ± 0.25	1 and 2	0.002	/	/
	2	0.52 ± 0.23	0.18 ± 0.16^bcd^	0.37 ± 0.24	0.41 ± 0.29				
						2 and 3	0.008	/	/
	3	0.41 ± 0.24	0.40 ± 0.27	0.37 ± 0.24	0.37 ± 0.28				

Group 1: Dry eye without AE, Group 2: Dry eye with AE, Group 3: Pre-clinical dry eye with AE

^a^ There was a significant difference compared with baseline (*P* < 0.05)

^b^ There was a significant difference compared with Group 1 (*P* < 0.05)

^c^ There was a significant difference compared with Group 3 (*P* < 0.05)

^d^ There were significant differences compared with the other time point (*P* < 0.05)

*TMH* tear meniscus height, *F-NITBUT* first non-invasive tear breakup time, *A-NITBUT* average non-invasive tear breakup time, *LLT* lipid layer thickness, *PBR* partial blink rate

### Changes in visual acuity before and after AE

Visual acuity in dry eye with AE group improved significantly at 0 and 30 min after AE compared to baseline (*P* = 0.017, *P* = 0.021) (Table 4).

**Table 4:** Comparison of visual acuity before (baseline) and after AE

Group	Time				*P v*alue			
	Baseline	0 min	30 min	60 min		Group × Time	Group main effect	Time main effect
1	83.4 ± 9.3	83.6 ± 9.1	83.4 ± 9.4	83.6 ± 9.4	1 and 2	0.034	/	/
2	83.2 ± 9.5	84.4 ± 9.6^a^	84.5 ± 9.4^a^	83.9 ± 9.2				
					2 and 3	0.038	/	/
3	83.7 ± 6.3	83.5 ± 6.3	83.4 ± 5.7	84.0 ± 5.6				

Group 1: Dry eye without AE, Group 2: Dry eye with AE, Group 3: Pre-clinical dry eye with AE

^a^ There was a significant difference compared with baseline (*P* < 0.05)

## Discussion

Studies of the effects of AE on dry eye mainly focused on the improvement of subjective symptoms in dry eye patients and the tear secretion in mice. In this study, AE promotes tear secretion and decreases the level of oxidative stress marker in tears and improves tear film stability in dry eye patients.

There was an increase in tear secretion at 30 min after AE in dry eye patients. AE excites the sympathetic nerves but inhibits the parasympathetic nerves. Parasympathetic nerves dominates the lacrimal gland which secrete tears [20]. The immediate impact of stimulation to the lacrimal gland by sympathetic excitement was likely to be limited. The sympathetic nerves in lacrimal gland are mainly located around the lacrimal gland acinar blood vessels [21], they can cause vasodilation and increase the secretion of electrolytes and water [22]. This might be the main cause of increased tear secretion in dry eye patients after AE. The lack of significant change in tear secretion in pre-clinical dry eye patients might be due to the limited effect of AE on secretory function of healthy lacrimal glands. This suggested that dry eye patients are more likely to benefit from AE.

Oxidative stress plays an important role in pathogenesis of dry eye and may be a potential treatment target for dry eye [23]. This study found that the oxidative stress marker 8-OHdG was significantly reduced after AE in dry eye patients. This was similar to the results in animal experiments that AE reduces 8-OHdG in tears of diabetic mice (with decreased tear secretion) [8]. Therefore, AE reduces the oxidative stress response on ocular surface. There were no significant changes in the other 5 tear compositions before and after AE, which might be related to short study period, long-term exercise could be different. Furthermore, the Schirmer I test without anesthetic mainly reflected the secretion of reflex tear, although the method of tear collection were consistent before and after AE, it was still possible to influence the results by diluting the basal tears.

In 2017, DEWS II emphasized the importance of tear film instability in the latest definition of dry eye [1]. The most commonly used tear film stability test clinically is tear breakup time. In dry eye patients, F-NITBUT and A-NITBUT were significantly longer after AE compared to baseline. This might be due to the changes of blinking parameters. Blinking is a fast eyelid movement and plays an important role in the dynamic balance of the ocular surface and tear film [24]. Blinking can effectively promote tear secretion, and replenish the tear film with tear from the inferior tear meniscus [25]. Blinking also makes the lipid layer to be evenly distributed on ocular surface. The lipid layer secreted by the meibomian gland prevent evaporation of tear and stabilize the tear film [26]. When the blinking is incomplete, the orbicularis muscle and the muscle of Riolan apply less squeezing force, resulting in insufficient driving force required for meibum secretion [27]. Moreover, the lipid layer becomes unevenly distributed on ocular surface thus affecting tear film stability. Clinical studies showed that the number of incomplete blinks was negatively associated with tear breakup time in dry eye patients [28]. In this study, the number of incomplete blinks and PBR at 10 min after AE decreased significantly compared to baseline, while the number of complete blinks increased significantly. This was helpful with tear secretion and more even distribution of tear film on ocular surface thus prolonging the tear breakup time and improving the stability of tear film.

In addition, visual acuity in dry eye patients improved after AE and maintained at least 30 min after AE. An intact and regular tear film is an important factor for high-quality retinal images. Dry eye patients with destabilized tear film show higher values of higher-order aberrations. This leads to excessive diopter variation before and after blinking causing visual fluctuation [29]. We speculated that the improvement of visual acuity in dry eye patients after AE was related to the change of higher-order aberration caused by the improvement of tear film stability. But to determine the effect of AE on visual acuity, higher-order aberration should be included in the observation, and influencing factors such as accommodative function and retinal function should be excluded.

There were also some limitations to this study. First, compared to the condition of constant temperature and humidity, the outdoor complex environment inevitably had an impact on results. However, the experiment reflected the true effects of outdoor exercise on dry eye in most people. Secondly, the age of study subjects were much younger than the average age of most dry eye patients, it is necessary to expand the sample size and rationalize the ratio of age and sex to obtain more comprehensive and reliable results. In addition, it is worth noting that it may be difficult and dangerous to apply AE in older adults. Finally, this study only observed the impact of AE just once on dry eye. Further observation is needed for the benefits of long-term regular AE on dry eye.

## Conclusion

In conclusion, this study showed that AE increases Schirmer I test, decreases the 8-OHdG in tears, increases F-NITBUT, A-NITBUT, the number of complete blinks, and decreases the number of incomplete blinks and PBR. This indicates AE promotes tear secretion and decreases the level of oxidative stress marker in tears and improves tear film stability in dry eye patients.

## Supplementary Information

**Additional file 1.** Ocular Surface Disease Index (OSDI). The OSDI is the most widely used questionnaire for dry eye clinical trials.

## Data Availability

The datasets used and/or analyzed during the current study are available from the corresponding author on reasonable request.

## Abbreviations

AE: Aerobic exercise
TMH: Tear meniscus height
F-NITBUT: First non-invasive tear breakup time
A-NITBUT: Average non-invasive tear breakup time
LLT: Lipid layer thickness
PBR: Partial blink rate
DEWS II: Dry Eye Workshop II
OSDI: The ocular surface disease index
8-OHdG: 8-hydroxy-2′-deoxyguanosine
OAHFA: (O-acyl)-ω-hydroxy fatty acids
MUC5AC: Mucin 5 subtype AC
