# Supplementary material for: Effects of aerobic exercise on tear secretion and tear film stability in dry eye patients
Source: BMC Ophthalmol. 2022 Jan 4;22:9. doi: 10.1186/s12886-021-02230-9 (PMC8725542; doi:10.1186/s12886-021-02230-9)
Supplement: Supplementary file 1 — Additional file 1. Ocular Surface Disease Index (OSDI). The OSDI is the most widely used questionnaire for dry eye clinical trials. [file 12886_2021_2230_MOESM1_ESM.pdf]

# Ocular Surface Disease Index© (OSDI©)[2]

Ask your patients the following 12 questions, and circle the number in the box that best represents each answer. Then, fill in boxes A, B, C, D, and E according to the instructions beside each.

| Have you experienced any of the following *during the last week*? | All of the time | Most of the time | Half of the time | Some of the time | None of the time |
|---|---|---|---|---|---|
| 1. Eyes that are sensitive to light? . . | 4 | 3 | 2 | 1 | 0 |
| 2. Eyes that feel gritty? . . . . . . . . . . . | 4 | 3 | 2 | 1 | 0 |
| 3. Painful or sore eyes? . . . . . . . . . . | 4 | 3 | 2 | 1 | 0 |
| 4. Blurred vision? . . . . . . . . . . . . . . . | 4 | 3 | 2 | 1 | 0 |
| 5. Poor vision? . . . . . . . . . . . . . . . . . | 4 | 3 | 2 | 1 | 0 |

**Subtotal score for answers 1 to 5** (A)

| Have problems with your eyes limited you in performing any of the following *during the last week*? | All of the time | Most of the time | Half of the time | Some of the time | None of the time | N/A |
|---|---|---|---|---|---|---|
| 6. Reading? . . . . . . . . . . . . . . . . . . . . | 4 | 3 | 2 | 1 | 0 | N/A |
| 7. Driving at night? . . . . . . . . . . . . . . | 4 | 3 | 2 | 1 | 0 | N/A |
| 8. Working with a computer or bank machine (ATM)? . . . . . . . . . . | 4 | 3 | 2 | 1 | 0 | N/A |
| 9. Watching TV? . . . . . . . . . . . . . . . . | 4 | 3 | 2 | 1 | 0 | N/A |

**Subtotal score for answers 6 to 9** (B)

| Have your eyes felt uncomfortable in any of the following situations *during the last week*? | All of the time | Most of the time | Half of the time | Some of the time | None of the time | N/A |
|---|---|---|---|---|---|---|
| 10. Windy conditions? . . . . . . . . . . . . . | 4 | 3 | 2 | 1 | 0 | N/A |
| 11. Places or areas with low humidity (very dry)? . . . . . . . . . . . | 4 | 3 | 2 | 1 | 0 | N/A |
| 12. Areas that are air conditioned? . . . | 4 | 3 | 2 | 1 | 0 | N/A |

**Subtotal score for answers 10 to 12** (C)

**Add subtotals A, B, and C to obtain D**
**(D = sum of scores for all questions answered)** (D)

**Total number of questions answered**
**(do not include questions answered N/A)** (E)

**Please turn over the questionnaire to calculate the patient's final OSDI© score.**

## Evaluating the OSDI© Score[1]

The OSDI© is assessed on a scale of 0 to 100, with higher scores representing greater disability. The index demonstrates sensitivity and specificity in distinguishing between normal subjects and patients with dry eye disease. The OSDI© is a valid and reliable instrument for measuring dry eye disease (normal, mild to moderate, and severe) and effect on vision-related function.

## Assessing Your Patient's Dry Eye Disease[1, 2]

**Use your answers D and E from side 1 to compare the sum of scores for all questions answered (D) and the number of questions answered (E) with the chart below.* Find where your patient's score would fall. Match the corresponding shade of red to the key below to determine whether your patient's score indicates normal, mild, moderate, or severe dry eye disease.**

| Number of Questions Answered (E from Side 1) \ Sum of Scores for All Questions Answered (D from Side 1) | 5 | 10 | 15 | 20 | 25 | 30 | 35 | 40 | 45 | 48 |
|---|---|---|---|---|---|---|---|---|---|---|
| 12 | 10.4 | 20.8 | 31.3 | 41.7 | 52.1 | 62.5 | 72.9 | 83.3 | 93.8 | 100.0 |
| 11 | 11.4 | 22.7 | 34.1 | 45.5 | 56.8 | 68.2 | 79.5 | 90.9 | 100.0 | |
| 10 | 12.5 | 25.0 | 37.5 | 50.0 | 62.5 | 75.0 | 87.5 | 100.0 | | |
| 9 | 13.9 | 27.8 | 41.7 | 55.6 | 69.4 | 83.3 | 97.2 | | | |
| 8 | 15.6 | 31.3 | 46.9 | 62.5 | 78.1 | 93.8 | 100.0 | | | |
| 7 | 17.9 | 35.7 | 53.6 | 71.4 | 89.3 | 100.0 | | | | |
| 6 | 20.8 | 41.7 | 62.5 | 83.3 | 100.0 | | | | | |
| 5 | 25.0 | 50.0 | 75.0 | 100.0 | | | | | | |
| 4 | 31.3 | 62.5 | 93.8 | | | | | | | |
| 3 | 41.7 | 83.3 | | | | | | | | |
| 2 | 62.5 | | | | | | | | | |
| 1 | | | | | | | | | | |

*Values to determine dry eye severity calculated using the OSDI© formula.

$$\text{OSDI©} = \frac{\text{(sum of scores)} \times 25}{\text{(\# of questions answered)}}$$

**Sum of Scores for All Questions Answered (D from Side 1)**

**Normal** **Mild** **Moderate** **Severe**

**Patient's Name:** ______________________ **Date:** ______________

**How long has the patient experienced dry eye disease?** ______________________

**Eye Care Professional's Comments:** ______________________


1. Data on file, Allergan, Inc.
2. Schiffman RM, Christianson MD, Jacobsen G, Hirsch JD, Reis BL. Reliability and validity of the Ocular Surface Disease Index. *Arch Ophthalmol.* 2000;118:615-621


Copyright © 1995, Allergan
